# The relation between parietal GABA concentration and numerical skills

**DOI:** 10.1038/s41598-021-95370-3

**Published:** 2021-09-03

**Authors:** George Zacharopoulos, Francesco Sella, Uzay Emir, Roi Cohen Kadosh

**Affiliations:** 1 Department of Experimental Psychology, Wellcome Centre for Integrative Neuroimaging, University of Oxford, Oxford, UK; 2 Department of Psychology, Swansea University, Swansea, UK; 3 Centre for Mathematical Cognition, Loughborough University, Loughborough, UK; 4 School of Health Sciences, College of Health and Human Sciences, Purdue University, West Lafayette, IN 47907-2051 USA; 5 School of Psychology, University of Surrey, Guildford, UK

**Keywords:** Human behaviour, Cognitive neuroscience

## Abstract

Several scientific, engineering, and medical advancements are based on breakthroughs made by people who excel in mathematics. Our current understanding of the underlying brain networks stems primarily from anatomical and functional investigations, but our knowledge of how neurotransmitters subserve numerical skills, the building block of mathematics, is scarce. Using ^1^H magnetic resonance spectroscopy (N = 54, 3T, semi-LASER sequence, TE = 32 ms, TR = 3.5 s), the study examined the relation between numerical skills and the brain’s major inhibitory (GABA) and excitatory (glutamate) neurotransmitters. A negative association was found between the performance in a number sequences task and the resting concentration of GABA within the left intraparietal sulcus (IPS), a key region supporting numeracy. The relation between GABA in the IPS and number sequences was specific to (1) parietal but not frontal regions and to (2) GABA but not glutamate. It was additionally found that the resting functional connectivity of the left IPS and the left superior frontal gyrus was positively associated with number sequences performance. However, resting GABA concentration within the IPS explained number sequences performance above and beyond the resting frontoparietal connectivity measure. Our findings further motivate the study of inhibition mechanisms in the human brain and significantly contribute to our current understanding of numerical cognition's biological bases.

## Introduction

Several scientific, engineering, and medical advancements are based on breakthroughs made by people who excel in mathematics (e.g., Archimedes, Newton, and Einstein). Numerical cognition is complex, and it is thought to consist of different components^1^. For example, a distinction can be drawn between (1) the representation of numerical information (factual knowledge) and (2) the manipulation of numerical information (procedural knowledge, e.g., cognitive manipulation of magnitudes), and these two categories were empirically shown to be double-dissociated in the case of developmental dyscalculia^2^. Moreover, further evidence exists supporting the premise that numerical cognition is componential. First, several acalculic patients, as a result of brain damage, show selective impairments in some components of numeracy (e.g., knowledge of numerical facts or comparing the relative size of numbers) but not in others (e.g., reading or writing numbers)^3,4^. Second, large individual differences were documented in healthy adults even in intuitively similar numerical components^5^. Third, studies employing factor analyses resulted in disparate components including (1) numerical facility, (2) mathematical reasoning^6^, (3) dot counting^7^, and (4) numerical estimation^8^.

Apart from the cognitive investigation, neuroimaging studies examined how numerical components are implemented at the neurobiological level. For example, a meta-analysis study has shown that arithmetic problem-solving recruits mainly the frontal and parietal lobes^9^. However, multiplication recruits the right hemisphere primarily, while addition recruits mainly the contralateral hemisphere, and solving subtraction problems is subserved by a bilateral activity^9^.

Similarly, other studies identified similar brain regions and networks underpinning numerical cognition with a special focus on the frontoparietal networks which are responsible for the representation and manipulation of numerical information over a short period (e.g., seconds)^10,11^. These networks have a significant overlap with the widely studied working memory systems^12,13^. The intraparietal sulcus (IPS) and the lateral prefrontal cortex are central seeds in these networks, and thus are particularly relevant for numerical cognition as documented in the prior extensive brain imaging work^9,14–16^. A recent meta-analysis using 57 neuroimaging papers revealed that frontal and parietal lobes, including different parietal subregions, support numerical representations^11^. Also, frontal regions were demonstrated to subserve magnitude representations, thus suggesting that both frontal and parietal regions are crucial for number processing. A more recent investigation directly examined the IPS's role in representing and manipulating numerical information and showed that anterior IPS is involved in encoding and that posterior parietal regions such as parietal lobules construct a working memory representation or orient spatial attention during number comparison^17^.

Despite these advancements, our knowledge of how the concentration of the brain’s major inhibitory (GABA) and excitatory (glutamate) neurotransmitters relate to numerical cognition has not yet been directly investigated. Unlike other neurochemicals, GABA and glutamate are abundant in the brain and directly affect brain activity, including regional blood-oxygen-level-dependent (BOLD) signal and brain connectivity in several networks including the default mode and the motor^18–22^. Importantly, recent studies demonstrated the role of glutamate and GABA in cortical excitation and inhibition, including in a single case of a mathematical prodigy, and in perceptual training, visual perception, attention, and cognitive skill acquisition^23–32^. These prior findings provide the motivation to examine the role of GABA and glutamate concentrations in tracking individual variation in numerical cognition. Therefore, the primary aim of the present study was to examine the neurotransmitter bases of several numerical tests involving rule-identification, reasoning, fluency, and knowledge of numerical facts. This would help address the question of which of those aspects of numerical cognition are associated with neurotransmitters. To this end, single-voxel proton magnetic resonance spectroscopy (^1^H-MRS) was employed to examine the capacity of GABA and glutamate concentration within key numerical parietal and frontal regions in explaining the variance in the performance of various numerical tasks (numerical operations, mathematical reasoning, tempo test, number sequences, computational estimation, numerical agility) in a group of young adults. Given that both left and right frontoparietal regions were shown to underpin numerical processing^10,33,34^, and since we did not aim to interrogate the effect of brain laterality in the present study, we merely focused on left frontoparietal regions to keep the duration of the study to an acceptable length. Given the well-established role of frontoparietal brain connectivity in numerical cognition and the well-documented impact of neurochemical concentration on resting brain connectivity, we additionally employed resting functional magnetic resonance imaging (fMRI) to examine the complementary role of frontoparietal connectivity^10,19,20,22^. Overall, our aims were to (1) examine the relation between numerical cognition and resting neurochemical concentration within frontal and parietal regions and to (2) investigate how these relations depend on resting frontoparietal brain connectivity.

## Results

### Descriptive statistics and correlations between the cognitive tests

As a first step, we examined the behavioural results. The descriptive statistics of our cognitive tests can be seen in Table 1. To assess whether performance in the aforementioned cognitive tests is significantly related to each other we performed bivariate correlations, and as can be seen in Table 2 there was a generally positive relation between them.

**Table 1:** Descriptive statistics, displayed in the first row, of the cognitive measures.

	Range	Minimum	Maximum	Mean	SD	Variance	Skewness	SE	Kurtosis	SE
Numerical operations	0.11	0.89	1.00	0.95	0.03	0.00	− 0.39	0.33	− 0.14	0.64
Mathematical reasoning	0.10	0.90	1.00	0.96	0.03	0.00	− 0.50	0.33	− 0.76	0.64
Number sequences	0.81	0.00	0.81	0.40	0.17	0.03	0.12	0.33	0.21	0.64
Tempo test	0.74	0.67	1.41	0.97	0.18	0.03	0.83	0.33	0.21	0.64
Computational estimation	0.90	0.00	0.90	0.43	0.19	0.04	0.29	0.32	-0.23	0.64
Numerical agility	0.80	0.00	0.80	0.50	0.18	0.03	− 0.44	0.33	− 0.12	0.64
Matrix reasoning	0.23	0.73	0.97	0.84	0.06	0.00	− 0.10	0.33	− 0.52	0.64

SD, standard deviation; SE, standard error.

**Table 2:** Correlation matrix depicting the correlation coefficients between the numerical and matrix reasoning tests.

	Numerical operations	Mathematical reasoning	Number sequences	Tempo test	Computational estimation	Numerical agility	Matrix reasoning
Numerical operations	1	.316*	.426**	0.242	0.261	.288*	0.237
Mathematical reasoning		1	.359**	.504**	.427**	0.246	.289*
Number sequences			1	.309*	.349*	0.218	0.180
Tempo test				1	.427**	.409**	.262
Computational estimation					1	0.201	.312*
Numerical agility						1	0.107
Matrix reasoning							1

*< 0.05 level (2-tailed), **< 0.01 level (2-tailed), uncorrected.

### GABA within the IPS is negatively associated with number sequences performance

**Figure 1:**
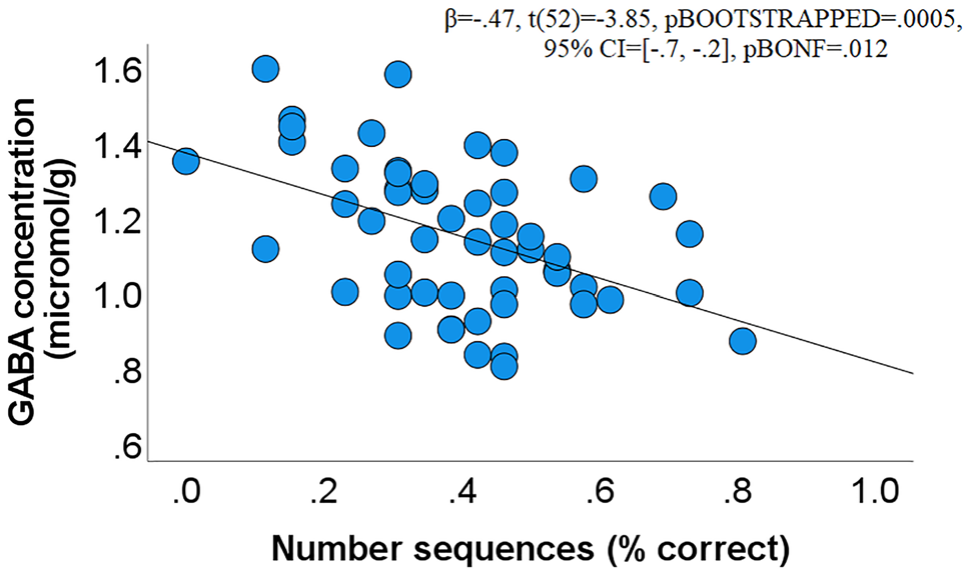
Scatterplot depicting a negative association between the accuracy in the number sequences subtest of the numerical aptitude test and the concentration of GABA within the left IPS (derived from Eq. 2).

We then examined the association between neurotransmitters and numerical performance. We detected a significant association only between the number sequences and the concentration of GABA within the left IPS (β = − 0.47, t(52) =  − 3.85, pBOOTSTRAPPED = 0.0005, 95% CI = [− 0.7, − 0.2], pBONF = 0.012, Fig. 1). This finding was present even after controlling for age (β = − 0.48, t(51) =  − 3.82, pBOOTSTRAPPED = 0.0003, 95% CI = [− 0.7, − 0.3]), gender (β = − 0.41, t(51) =  − 3.4, pBOOTSTRAPPED = 0.003, 95% CI = [− 0.6, − 0.1]), or general intelligence as assessed with matrix reasoning (β = − 0.49, t(50) = − 4.05, pBOOTSTRAPPED = 0.0001, 95% CI = [− 0.7, − 0.3]). The same analyses using the relative neurotransmitter method (/tCr) yielded the same results (β = − 0.41, t(52) = − 3.25, pBOOTSTRAPPED = 0.0009, 95% CI = [− 0.6, − 0.2], pBONF = 0.022). Please see Table 3 for all effect sizes (correlation coefficients, r).

**Table 3:** Correlation matrix (correlation coefficients) depicting the associations between the numerical measures and neurotransmitter concentrations.

	Numerical operations	Mathematical reasoning	Number sequences	Tempo test	Computational estimation	Numerical agility
**Frequentist statistics**						
GABA MFG	.076	− .036	− .059	− .030	− .271	.165
Glutamate MFG	− .018	.265	.172	.233	− .005	.202
GABA IPS	− .104	− .128	− .471**	− .107	− .136	.011
Glutamate IPS	− .244	.108	− .190	.041	− .002	.086

**0.01 level (2-tailed), *0.05 level (two-tailed) uncorrected.

### IPS GABA and number sequences association is cognitively specific

To delve more into the underlying structure of our six mathematical measures, we ran principal components analyses where a single factor was extracted (see Methods section) which was not significantly associated to IPS GABA (β = − 0.2, t(50) = − 1.45, pBOOTSTRAPPED = 0.16, 95% CI = [− 0.48, 0.07]). We then regressed out the variance of this “general mathematical ability” factor from the number sequences, and these resulting residuals were significantly predicted by IPS GABA (β = − 0.41, t(50) = − 3.2, pBOOTSTRAPPED = 0.004, 95% CI = [− 0.6, − 0.1]). This set of results shows that IPS GABA tracks individual variation in number sequences and not general mathematical ability.

### IPS GABA and number sequences association is regionally and neurochemically specific

The above analyses allowed us to establish an association between the concentration of GABA within the left IPS and performance in the number sequences task. To test the neurochemical and anatomical specificity of our findings, we employed a multiple regression model predicting number sequences based on three predictors (1) GABA within the IPS (β = − 0.5, t(45) = − 3.36, pBOOTSTRAPPED = 0.001, 95% CI = [− 0.75, − 0.21]), (2) GABA within the MFG (β = − 0.005, t(45) = − 0.04, pBOOTSTRAPPED = 0.97, 95% CI = [− 0.25, 0.22]), (3) glutamate within the IPS (β = − 0.06, t(45) = − 0.5, pBOOTSTRAPPED = 0.58, 95% CI = [− 0.3, 0.2]), and found that only the GABA within the IPS predictor was significant.

### Left frontoparietal connectivity is positively associated with number sequences performance

**Figure 2:**
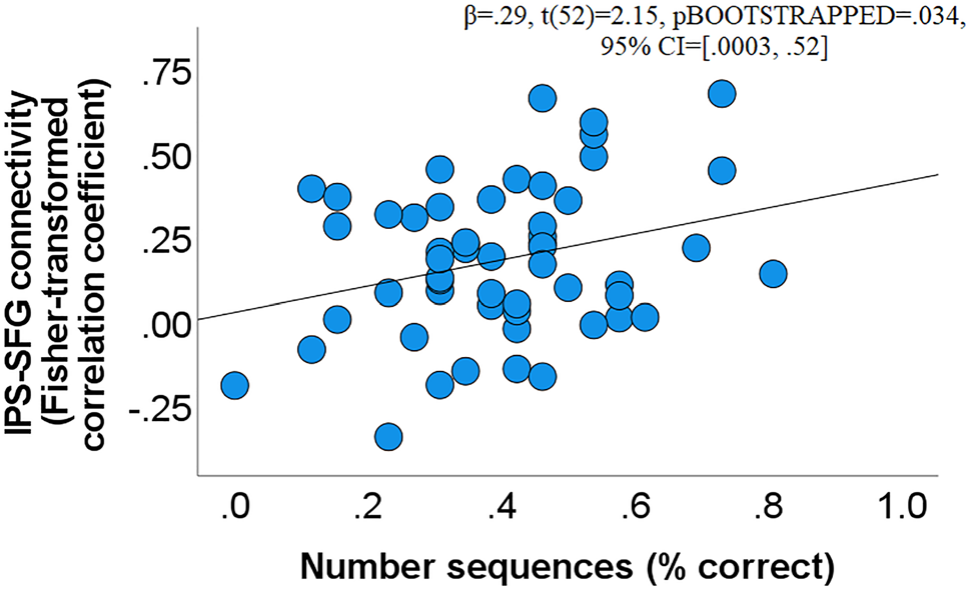
Scatterplot depicting a positive association between the accuracy in the number sequences test and the connectivity between the left IPS and the left SFG.

After establishing the association between GABA concentration within the IPS and number sequences, we employed resting fMRI to investigate whether left frontoparietal connectivity is related to number sequences. To this end, we checked four connectivity pairs, (1) left IPS- left SFG, (2) left IPS- left MFG, and (3) left IPS- left IFG pars triangularis, and (4) left IPS- left IFG pars opercularis. The strongest association was detected for the first pair (β = 0.29, t(52) = 2.15, pBOOTSTRAPPED = 0.034, 95% CI = [0.0003, 0.52], Fig. 2). The results for the other three left frontal regions were as follows: MFG (β = 0.017, t(52) = 0.17 pBOOTSTRAPPED = 0.88, 95% CI = [− 0.22, 0.24]), IFG pars triangularis (β = 0.04, t(51) = 0.26, pBOOTSTRAPPED = 0.8, 95% CI = [− 0.25, 0.34]), IFG pars opercularis (β = 0.13, t(52) = 0.92, pBOOTSTRAPPED = 0.41, 95% CI = [− 0.17, 0.42]).

### IPS GABA explains number sequences performance above and beyond left frontoparietal connectivity

**Figure 3:**
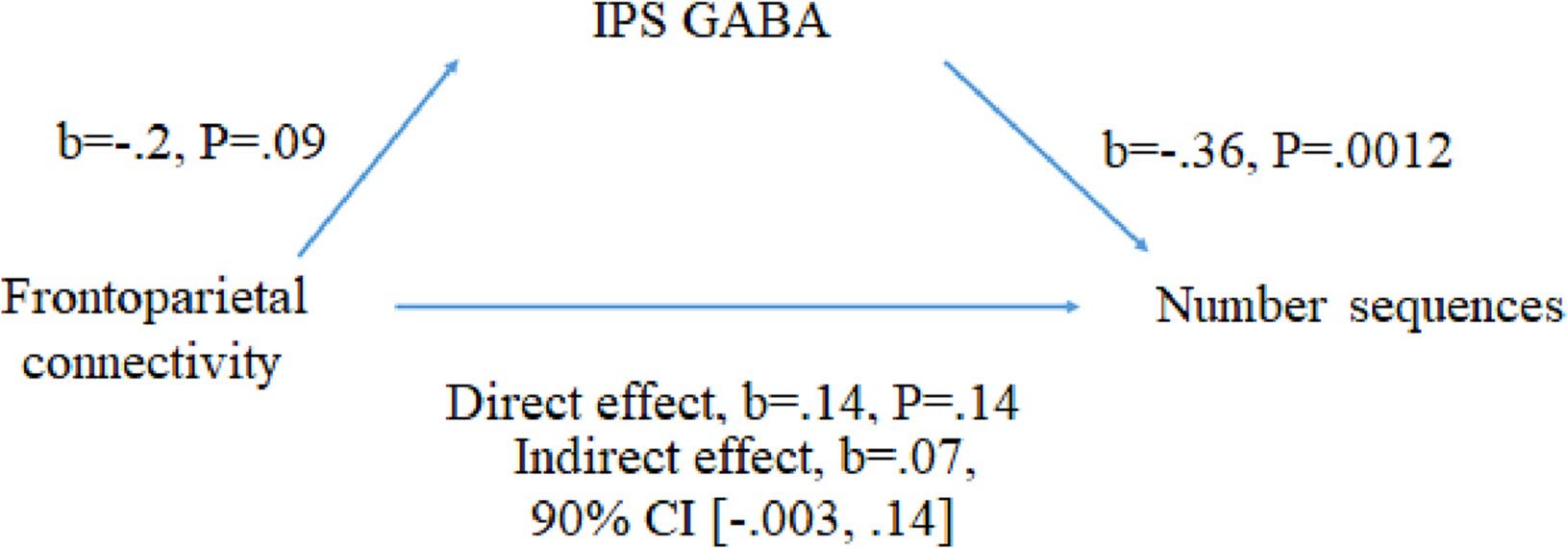
A trend showing that frontoparietal connectivity predicts number sequences by the mediation of IPS GABA concentration.

The relation between GABA within the IPS and left IPS-SFG connectivity showed a negative trend (β = − 0.23, t(52) = − 1.71, pBOOTSTRAPPED = 0.09, 95% CI = [− 0.4, 0.05]). To examine the interdependence of left frontoparietal connectivity and IPS GABA in tracking number sequences performance we employed a multiple regression featuring both predictors. This analyses showed that GABA IPS was still significant (β = − 0.43, t(51) =  − 3.44, pBOOTSTRAPPED = 0.002, 95% CI = [− 0.57, − 0.15]) while left frontoparietal connectivity was not (β = 0.19, t(51) = 1.50, pBOOTSTRAPPED = 0.14, 95% CI = [− 0.06, 0.31]). Since frontoparietal connectivity is associated with number sequences (step 1), and IPS GABA (step 2), and since IPS GABA still predicts number sequences after controlling for frontoparietal connectivity (step 3), then the IPS GABA can be viewed as a mediator on the relation between frontoparietal connectivity and number sequences^36^, but a formal mediation test showed that this was not the case even though there was a trend (Fig. 3, CI of the indirect effect = [− 0.003 0.14]). Taken together our results suggest that resting IPS GABA tracks individual variation in number sequences above and beyond resting left frontoparietal connectivity.

## Discussion

The present study investigated the relation between the brain’s major inhibitory and excitatory neurotransmitters, GABA and glutamate, and numerical cognition as assessed using several key numerical measures. We found a negative association between a complex numerical measure, the number sequences, and the amount of GABA within the left IPS. This was followed by (1) a cognitive, (2) a neurotransmitter, and (3) a regional specificity supporting the explicitness of our finding. Subsequently, we demonstrated a positive association between left frontoparietal connectivity and number sequences performance. Lastly, we show that the resting GABA concentration within the IPS is a stronger and independent predictor of number sequences above and beyond the observed resting frontoparietal connectivity.

The main finding of the present study was the negative association between GABA concentration in the left IPS and number sequences performance. The number sequences task required the identification of a logical rule that regulates the relations between numbers in a sequence. The process of identifying such a rule involves a reiterative formulation and testing of hypotheses along with continuously performing calculations. As an example, testing the “increasing difference” hypothesis in the sequence 1 3 6 10 15 21 _, one would need to perform several subtractions (e.g., 3–1, 6–3, 10–6, etc.), while keeping active the partial results in the aforementioned frontoparietal networks, and verify whether the hypothesized rule is respected (i.e., differences increase by one unit)^10^.

Notably, the number sequences task contains underlying cognitive components also assessed in other administered tasks. First, numerical procedures are included in numerical operations, mathematical reasoning, and the tempo test. Second, numerical flexibility is included in other tests like the numerical agility test. Third, the seek for a logical rule is also measured outside the numerical domain using the matrix reasoning task. However, the logical rule in the number sequences is numeric unlike the one in the matrix reasoning task. Taken together, we conclude that the GABA concentration in the left IPS tracks a unique and specific set of abilities concerning the identification of a logical rule particularly in the numerical domain that additionally involves numerical flexibility.

An open question for future research is the delineation of how IPS GABA affects the numerical brain at the network level. The biological significance of GABA modulation during rest was established in several settings. First, it was shown that variation in baseline GABA level, as measured by MRS, correlates with blood oxygenation-dependent (BOLD) fMRI signal in the visual, somatosensory and anterior cingulate cortex regions^37–40^. Moreover, GABA concentration during rest was additionally associated to several sensory and cognitive functions, including monocular deprivation, perceptual training, visual perception, attention and cognitive skill acquisition^24,25,27,30,32^. Here, we extend the biological significance of regional resting GABA concentration in tracking individual variation in number sequences performance. Given the well-established role of frontoparietal brain connectivity in numerical cognition and the well-documented impact of neurochemical concentration on resting brain connectivity, we subsequently utilized resting fMRI to examine the role of frontoparietal connectivity. Our results suggest that frontoparietal connectivity does not uniquely contribute to number sequences once GABA concentration is taken into account, while GABA concentration can predict number sequences above and beyond frontoparietal connectivity. Therefore, it is currently unknown how IPS GABA affects the numerical brain network level. Future studies can delve more into the role of IPS GABA at the brain network level by linking IPS GABA concentration to frontal and parietal regional activations obtained in a task-based fMRI setting. This multi-modal approach has the potential to offer further insights concerning the neurofunctional relevance of IPS GABA in the context of numerical cognition at the brain network level. In particular, if the relation between IPS GABA and number sequences performance is mediated by the individual variation in task-based brain activity, this set of findings would shed some light on the neurodynamic effect of IPS GABA at the brain network level.

By interrogating regional specificity, we found that parietal but not frontal GABA, and parietal GABA but not parietal glutamate, was related to number sequences. Why is parietal but not frontal GABA (and not glutamate) an important index of numerical cognition? Two competing hypotheses have been proposed concerning neurochemical mechanisms across the brain^41^. The first postulates that neurochemical concentration is uniquely determined in each region and each individual by genetic and environmental influences, thus neurochemicals in different regions may exert different influences on sensory and cognitive processing^30,42–44^. The second theory postulates that neurochemical concentration across different regions may be very similar due to common embryonic origins or shared subcortical projections^45–49^. Such neurochemical selectivity was previously demonstrated in other domains of neuroscience including visual perception and attention^27,41^. Our results support both a regional and a neurochemical selective influence on number sequences, therefore, supporting the first theory. Overall, unlike the IPS, the neurochemical contribution of MFG did not track numerical performance. Meta-analyses suggested a hierarchical contribution of prefrontal cortex in numerical cognition^9^. Namely, the inferior frontal gyrus was typically engaged in relatively simple numerical tasks, while the MFG is involved in more complex tasks which require several procedural steps or increase storage load^9^. This role of MFG likely reflects shared links to working memory which is behaviourally related to numerical performance^50,51^ and supported by the prefrontal cortex^52,53^. Indeed, a recent study employing functional MRS found elevated glutamate levels in dorsolateral prefrontal cortex during the execution of a 2-back task compared to passive visual fixation^54^. Given this contribution of MFG in demanding computations that mirror the computations underlying the present cognitive tasks, one potential reason we did not find an association between MFG neurotransmitter levels and performance may be accounted for the fact that neurotransmitter levels were measured at baseline rather than during the execution of the numerical tasks which is indeed a limitation of the present study. This consequently prevents us from drawing conclusions regarding the task-based contribution of neurotransmitter and frontoparietal connectivity measures which are likely to differ compared to resting contributions.

Another limitation of the present study is common to other MRS studies and relates to the difficulty of distinguishing between intracellular and extracellular neurotransmitter concentrations or even a portion of these based on the MRS signal alone^55^. Consequently making direct inferences of cortical excitability/inhibition based on the neurotransmitter concentrations alone should be done with caution. Similar limitations concerning the exact nature of the neural signal are not specific to MRS but exist with other modalities e.g., diffusion and structural MRI^56,57^. Therefore, our finding demonstrates the pressing need for a multi-modal imaging approach for examining GABAergic mechanisms involved in numeric cognition.

In sum, by investigating numerical cognition with MRS, we identified a specific neurotransmitter, GABA in the IPS, a key brain region involved in numeracy, that is associated to a number sequences task. Our findings shed light on the possible mechanisms that are involved in individual differences in numeracy. Given the involvement of GABA in neuroplasticity^58^, our findings raise the question of whether such mechanisms are involved in the learning and development of numerical skills. These findings also motivate future examination of the underlying causality mechanisms which could be assessed using non-invasive brain stimulation approaches^25,59–61^.

## Methods

### Participants

We recruited 54 university first-year undergraduate participants (21 females, Mean age: 18.89, SD: 0.62). The completion of the imaging session lasted (~ 60 min), and the completion of the cognitive-behavioural testing lasted (~ 60 min); these sessions were part of a more extensive battery that included several other cognitive and behavioural assessments. All imaging data were acquired on a single scanning session. Because the magnetic resonance spectroscopy sequence we utilized measures baseline-level neurochemical concentration, participants watched a movie during the acquisition rather than performing the numerical tasks. During the “eyes-open” resting fMRI scan, participants were asked to fixate on a white cross on a black background. All participants were predominantly right-handed, as measured by the Edinburgh Handedness Inventory^62^. We confirmed via a questionnaire that they were free of current or past neurological, psychiatric or learning disability or condition that might affect cognitive or brain functioning. As compensation, participants received £50. Informed written consent was obtained. All methods were carried out in accordance with relevant guidelines and regulations. The study was approved by the University of Oxford’s Medical Sciences Interdivisional Research Ethics Committee (MS-IDREC-C2_2015_016).

### MRI data acquisition and preprocessing

All MRI data were acquired on a 3 T Siemens MAGNETOM Prisma MRI System equipped with a 32 channel receive-only head coil.

### Structural MRI

Anatomical high-resolution T1-weighted scans were acquired consisting of 192 slices, repetition time (TR) = 1900 ms; echo time (TE) = 3.97 ms; voxel size = 1 × 1 × 1 mm).

### Magnetic resonance spectroscopy

**Figure 4:**
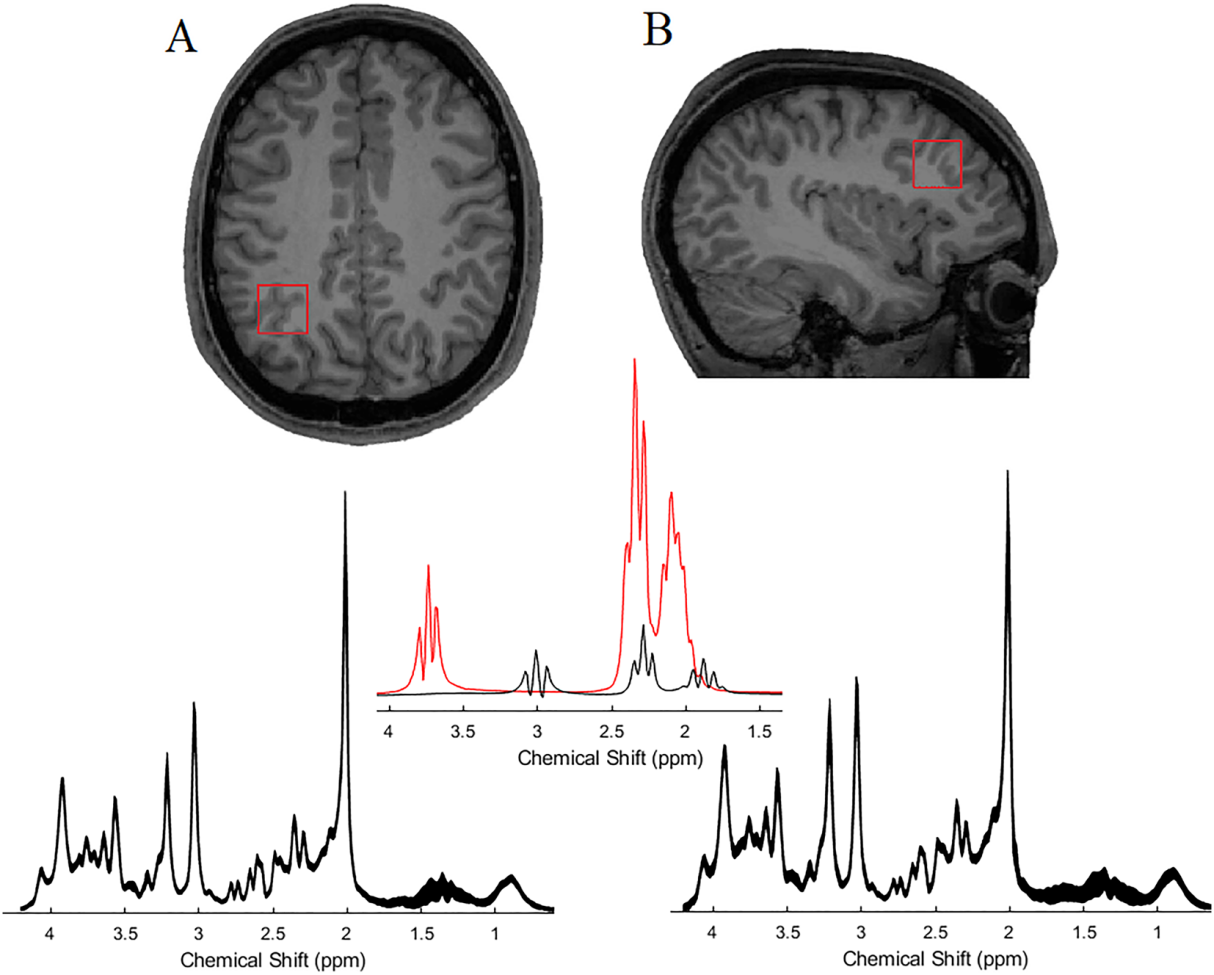
The positions displayed in a T1-weighted image for (**A**) IPS, (**B**) MFG, are shown on axial and sagittal slices, respectively. Below each panel, the mean spectrum from our sample of each region is shown. The line thickness corresponds to ± 1 standard deviation from the mean. The middle panel represents fit spectra for glutamate (red) and GABA (black). (Modified with the permission from^35^).

Spectra were measured by semi-adiabatic localization using an adiabatic selective refocusing (semi-LASER) sequence (TE = 32 ms; TR = 3.5 s; 32 averages)^63,64^ and variable power RF pulses with optimized relaxation delays (VAPOR), water suppression, and outer volume saturation. Unsuppressed water spectra acquired from the same volume of interest were used to remove residual eddy current effects and to reconstruct the phased array spectra with MRspa (https://www.cmrr.umn.edu/downloads/mrspa/. Two 20 × 20 × 20 mm voxels of interest were manually placed centred on the left IPS (IPS, Fig. 4A) and centred on left middle frontal gyrus (henceforth, MFG, Fig. 4B) on axial, sagittal and coronal structural slices while the participant lay down in the MR scanner. Acquisition time per voxel was 10–15 min including sequence planning and shimming.

MRS neurotransmitters were quantified with the LCmodel^65^, using a basis set of simulated spectra generated based on previously reported chemical shifts and coupling constants based on a VeSPA (versatile simulation, pulses, and analysis) simulation library^66^. Simulations were performed using the same RF pulses and sequence timings as in the 3T system described above. Absolute neurotransmitter concentrations were extracted from the spectra using a water signal as an internal concentration reference. The exclusion criteria for data were Cramer-Rao lower bounds (CRLB, the estimated error of the neurotransmitter quantification) > 50% were classified as not detectable. Additionally, we excluded cases with an SNR beyond 3 standard deviations and a concentration value (or a cognitive or a connectivity value, see sections below) that was beyond 3 standard deviations. Raw metabolite-to-water amplitude ratio neurotransmitter concentrations were then corrected using the structural properties of the selected regions. Namely, we segmented the images into different tissue classes, including grey matter (GM), white matter (WM), and cerebrospinal fluid (CSF) using the SPM12 segmentation facility. Next, we calculated the number of GM, WM, and CSF voxels within the two masks of interest separately around the left middle frontal gyrus (MFG) and the left intraparietal sulcus (IPS) in native space. Subsequently, we divided these six numbers (GM, WM, and CSF for IPS and MFG) by the total number of GM, WM, and CSF voxels creating the corresponding GM, WM, and CSF fraction values per participant and region. As a final computation step, we corrected the raw metabolite-to-water amplitude ratio neurotransmitter values to these structural fractions using the following LCmodel^65^ computation, as can be seen in Eq. 1. The numbers in the equation (43,300, 55,556, and 35,880) represent the amount of water concentration in the white matter (35,880), grey matter (43,300) and CSF (55,556), and these values were selected based on the LCmodel manual recommendations.

1 $${\text{Tissue - corrected}}\,{\text{concentration}} = \left( {\left( {{43,3}00{/55,556}*{\text{GM}}\,{\text{fraction}} + {35,88}0{/55,556}*{\text{WM}}\,{\text{fraction}} + {1}*{\text{CSF}}\,{\text{fraction}}} \right){/}\left( {{1} - {\text{CSF}}\,{\text{fraction}}} \right)} \right)*{\text{Raw}}\,{\text{metabolite - to - water}}\,{\text{ amplitude}}\,{\text{ ratio}}\, {\text{neurotransmitter}}\,{\text{ concentration}}$$

To minimize the potential confounding effects T2-relaxation times we corrected the concentration from Eq. 1 based on the T2 of tissue water values as can be seen in Eq 2. Fully relaxed unsuppressed water signals were acquired at TEs ranging from 32 to 4040 ms (TR = 15 s) to water T2 values in each region (32 ms, 42 ms, 52 ms, 85 ms, 100 ms, 115 ms, 150 ms, 250 ms, 450 ms, 850 ms, 1650 ms, 3250 ms, 4040 ms). The transverse relaxation times (T2) of tissue water and percent CSF contribution to the region were obtained by fitting the integrals of the unsuppressed water spectra acquired in each region at different TE values with a biexponential fit^67^, with the T2 of CSF fixed at 740 ms and three free parameters: T2 of tissue water, the amplitude of tissue water, and amplitude of CSF water. These analyses were conducted on a subject-by-subject basis. Our analysis pipeline was also used previously in an overlapping cohort of participants^35^. We used the LCModel default output with the following parameters: ATTMET, ATTH2O, and WCONC, which in our control files were set as follows ATTMET = 32, ATTH2O = 1, WCONC = 55,555, PPMSHF = 0.0, PPMEND = 0.5, PPMST = 4.2, DKNTMN = 0.25, RFWHM = 2.5, SDDEGP = 5.00,SDDEGZ = 5.00, DOWS = TRUE, DOECC = FALSE, DELTAT = 0.0001666, NUNFIL = 2048, HZPPPM = 123.2571, DEGPPM = 0, DEGZER = 0.

2 $${\text{T2 - corrected}}\,{\text{ concentration}} = {\text{tissue - corrected }}\,{\text{concentration}}*{\text{exp}}\left( { - {\text{TE}}/{\text{T2}}} \right)$$

For triangulation purposes, we additionally calculated the metabolite ratios with respect to total creatine, which was calculated as the raw metabolite-to-water amplitude ratio neurotransmitter concentration referenced the concentration of total creatine (i.e., tCr, where tCr = creatine and phosphocreatine concentration).

### Resting fMRI

Functional images were acquired with a multi-band acquisition sequence (Multi-band accel. factor = 6, TR = 933 ms, TE = 33.40 ms, flip angle 64°, number of slices = 72, voxel dimension = 2 × 2 × 2 mm, number of volumes: 380). rsfMRI data were pre-processed and analyzed using the CONN toolbox (www.nitrc.org/projects/conn, RRID: SCR_009550) in SPM12 (Wellcome Department of Imaging Neuroscience, Institute of Neurology, London, UK) using the default pre-processing pipeline “MNI-space direct normalization”^68^. Functional volumes were motion-corrected, slice-timed corrected, segmented, normalized to a standardized (MNI) template, spatially smoothed with a Gaussian kernel (8-mm FWHM) and a pass filtered (0.01 Hz to Inf). The low-pass portion of the filter (0.01 Hz) was used to reduce the physiological and noise components contributing to the low-frequency segment of the BOLD signal, the high-pass portion of the filter was set to Inf to additionally allow higher frequencies to be included (above 0.1 Hz) given our fast acquisition. It also increased the degrees of freedom improving the quality of denoising and connectivity estimation. We also accounted for physiological noise in the time-series by regressing out the confounding effects of white matter, CSF, realignment, and scrubbing that were automatically calculated in CONN. All frontal regions of interest (ROI) were defined based on the Harvard–Oxford Atlas (superior frontal gyrus, (SFG), middle frontal gyrus (MFG), and inferior frontal gyrus (IFG)), apart from the IPS which was defined based on the Dorsal Attention network atlas, both atlases are featured within the CONN toolbox. The reason we used a different atlas for the IPS is that this region is not featured in the other atlas. We calculated the (frontoparietal) ROI-to-ROI connectivity which we then added into the simple regression and multiple regression models. Instead of merely using the MFG, the choice of also including the IFG and SFG was based on the knowledge that several regions of the frontal gyrus are part of the frontoparietal networks involved in mathematical cognition^10^.

### Cognitive tests

Participants completed several tests assessing numerical skills: the numerical operations and the mathematical reasoning subtests of the Wechsler Individual Achievement Test (WIAT-II UK;^69^), the tempo test^70^, the number sequences subtest of the numerical aptitude test^71^, the computational estimation^72^ and the numerical agility^73^ tests.

The numerical operations subtest is composed of written numerical problems, which require the implementation of numerical procedures with minimal/absent time pressure.

The mathematical reasoning subtest is composed of mathematical problems, which require participants to create a mental model of the problem, extract relevant information, and then select and execute the appropriate operation^74^. We calculated the proportion of correct responses for the numerical operations and the mathematical reasoning subtests. We did not use the standardized scores as all participants were between 18 and 21 years of age.

The tempo test entails five columns (addition, subtraction, multiplication, division, and mixed), each composed of 40 numerical problems (e.g., 7 + 8 = ….). Each column is presented sequentially with the instruction to solve as many problems as possible within 60 s. The time constrains make the tempo test a widely used measure of numerical fluency. For the tempo test instead, we calculated the proportion of correct responses in the five columns, and then we divided it by the individual solving time divided by total time at disposal (i.e., 300 s). This calculation was particularly done to take into account the fact that several participants completed the columns within one minute.

The computational estimation task consists of ten numerical problems: five multiplications (e.g., 64.6 × 0.16) and five divisions (e.g., 0.76/0.89) which were presented simultaneously for one minute, and participants were asked to write down their best estimate for each numerical problem. Participants were explicitly told to estimate without calculating the exact answer. For each response, we calculated the proportion of its absolute deviation from the exact answer (the proportion of absolute deviation =|estimate-correct answer|/correct answer). When estimates differed from the correct answer by more than the value of the correct answer, such responses were deemed outliers and were replaced with ‘1’, as missing values. Therefore, ‘1’ was the maximum error score. Outliers were replaced rather than removed to avoid unfairly disadvantaging participants who misunderstood the task (e.g., by failing to notice the change in numerical sign from multiplication to division). Missing values were also replaced with ‘1’ rather than removed to avoid unfairly advantaging participants who decided not to complete the task and to spend more time on fewer calculations. Therefore, we decided to use this imputation method to avoid giving an advantage to slow responders. For instance, a given slow respondent may have provided three accurate estimates (i.e., a small deviation in proportion), while a given fast responder may have provided 10 less accurate estimates (i.e., large deviation in proportion). The calculation of average deviation would indicate a higher score for the slower than the fast responder. Therefore, by conversely giving a “1” to the missed responses of the slow participant somewhat balanced this discrepancy. To enhance the interpretability of the results we reversed the final computational estimation score by subtracting it from one, thus positive scores indicated higher performance.

In the numerical agility task, participants were asked to generate the number 24 from four presented numbers (for example with the numbers 7, 5, 5, 4 a solution could be (7–5/5) * 4). There were five problems in total, which were administered in order of difficulty. There was a two-minute time limit for each problem. The instructions made it clear that only the four basic operations were allowed, such that any individual having completed secondary school should be able to solve these problems. For each item, two points were awarded if the correct answer was achieved within one minute, one point was awarded if it was achieved within two minutes, and no points were awarded otherwise.

The number sequences subtest is composed of 26 complex number sequences reflecting a specific principle/rule. Participants had to complete the sequences by adding the last number of the sequence from a pool of five numbers at disposal (e.g., 1 3 6 10 15 21 _; Choices: 26 27 28 29 30). Participants had 15 min at disposal to solve as many sequences as they could. For this test, we calculated the proportion of correct responses. To assess the cognitive specificity of our associations, we also assessed the participants’ general cognitive ability using the matrix reasoning test^75^ which is a 30-item (of increasing difficulty) assessment tool which requires identifying a logical pattern in a sequence of visuospatial stimuli. The subtest is interrupted when the participant provides 3 consecutive wrong responses. We calculated the proportion of correct responses.

### Statistical analyses

To assess the association between neurotransmitter concentration and numerical performance, we employed bivariate correlation analyses, as well as multiple regressions. As can be seen in Table 4, some cognitive variables were not normally distributed. In these cases and when applicable we performed Spearman’s correlation (correlation analyses) and bootstrapping methods (multiple regression analyses), both of which are not sensitive to the assumption of normality. The bootstrapped P-values in the results section were obtained from 10,000 samples at 95% confidence intervals (CI) (SPSS v25)^76^. Our Bonferroni corrected P-value was 0.0021 derived from dividing our alpha value (0.05) by 24 [(4 brain measures (GABA MFG, GABA IPS, glutamate MFG, glutamate IPS)*6 numerical tasks)].

**Table 4:** Test of normality results table of the neurotransmitter and cognitive measures.

	Kolmogorov-Smirnova			Shapiro–Wilk		
	Statistic	*df*	Sig	Statistic	*df*	Sig
MFG GABA	0.088	49	.200*	0.980	49	0.572
IPS GABA	0.090	54	.200*	0.977	54	0.392
MFG glutamate	0.094	50	.200*	0.984	50	0.746
IPS glutamate	0.070	54	.200*	0.988	54	0.859
Tempo test	0.120	53	0.055	0.937	53	0.007
Numerical operations	0.166	54	0.001	0.941	54	0.010
Mathematical reasoning	0.189	54	0.000	0.916	54	0.001
Matrix reasoning	0.161	53	0.002	0.946	53	0.018
Number sequences	0.094	54	.200*	0.985	54	0.744
Numerical agility	0.179	53	0.000	0.950	53	0.027
Computational estimation	0.106	54	0.198	0.982	54	0.602
lIPS-lSFG	0.056	54	.200*	0.992	54	0.976
lIPS-lMFG	0.089	54	.200*	0.978	54	0.430
lIPS-lIFGtr	0.139	53	0.012	0.966	53	0.133
lIPS-lIFGop	0.079	54	.200*	0.974	54	0.284

l = left, SFG = superior frontal gyrus, MFG = middle frontal gyrus, IFGtr = inferior frontal gyrus pars triangularis, IFGop = inferior frontal gyrus pars opercularis, df = degrees of freedom, Sig. = P-value.

To delve more into the underlying structure of the six mathematical measures we performed principal component analyses. The observed correlation matrix was significantly different from an identity matrix as assessed with Bartlett’s test of sphericity (Approx. Chi-Square(15) = 62.08, P < 0.001) and the Kaiser–Meyer–Olkin measure of sampling adequacy was 0.79 which indicates that this set of variables are appropriate for component or factor analyses^77–79^. One factor was extracted which accounted for 45.46% of the variation in our measures. In particular, this factor’s association with each of the measures was as follows (tempo test, r_S_(50) = 0.70, P < 0.001; numerical operations, r_S_(50) = 0.57, P < 0.001; mathematical reasoning, r_S_(50) = 0.77, P < 0.001; number sequences, r(50) = 0.69, P < 0.001; numerical agility test, r_S_(50) = 0.56, P < 0.001; computational estimation, r(50) = 0.63, P < 0.001).
